# Utilization of screening and treatment for osteoporosis among stroke survivors

**DOI:** 10.3389/fendo.2022.1043863

**Published:** 2022-12-01

**Authors:** Chin-Hao Hsu, Sheng-Feng Sung, Hsin-Yi Yang, Wan-Ting Huang, Cheng-Yang Hsieh

**Affiliations:** ^1^ Division of Plastic Surgery, Department of Surgery, Ditmanson Medical Foundation Chia-Yi Christian Hospital, Chiayi City, Taiwan; ^2^ Division of Neurology, Department of Internal Medicine, Ditmanson Medical Foundation Chia-Yi Christian Hospital, Chiayi City, Taiwan; ^3^ Clinical Data Center, Department of Medical Research, Ditmanson Medical Foundation Chia-Yi Christian Hospital, Chiayi City, Taiwan; ^4^ Clinical Research Center, Department of Medical Research, Ditmanson Medical Foundation Chia-Yi Christian Hospital, Chiayi City, Taiwan; ^5^ Department of Neurology, Tainan Sin Lau Hospital, Tainan, Taiwan; ^6^ School of Pharmacy, Institute of Clinical Pharmacy and Pharmaceutical Sciences, College of Medicine, National Cheng Kung University, Tainan, Taiwan

**Keywords:** stroke, fracture, osteoporosis, bone mineral density, treatment

## Abstract

**Background:**

Stroke survivors are prone to osteoporosis and fractures. However, bone mineral density (BMD) testing and osteoporosis treatment were underutilized in patients with recent stroke. We aimed to examine whether stroke has an impact on the utilization of BMD testing and osteoporosis treatment as well as the determinants of their utilization in stroke patients using nationwide population-based data in Taiwan.

**Methods:**

We identified patients aged 55 years and older who were hospitalized for hemorrhagic or ischemic stroke as the stroke cohort, and age- and sex-matched patients hospitalized for reasons other than stroke, fracture, or fall as the non-stroke cohort. We used the Fine-Gray sub-distribution hazard competing risk regression model to determine the predictors for BMD testing and osteoporosis treatment.

**Results:**

A total of 32997 stroke patients and 32997 age- and sex-matched controls comprised the stroke and non-stroke cohorts, respectively. BMD testing and osteoporosis treatment were performed in 1.0% and 5.2% of the stroke patients, respectively, within one year after hospitalization while these measures were performed in 0.8% and 4.7% of the controls. Stroke patients were more likely to receive BMD testing (adjusted hazard ratio [HR] 1.33; 95% confidence interval [CI] 1.11–1.58) and osteoporosis treatment (adjusted HR 1.19; 95% CI 1.11–1.29). Female sex, osteoporosis, prior BMD testing, and low-trauma fractures after stroke increased the likelihood of using BMD testing and osteoporosis treatment whereas greater stroke severity reduced the likelihood of receiving both measures.

**Conclusions:**

Both BMD testing and osteoporosis treatment were underutilized among stroke survivors even though they had a higher chance of receiving both measures than non-stroke patients.

## Introduction

Stroke is the third leading cause of disability-adjusted life-year (DALY) loss and the global burden of stroke keeps increasing (1, 2). In the past 30 years, the worldwide absolute numbers of incident strokes and deaths from stroke have increased by 70% and 43%, respectively (3). In 2019, more than 100 million people were living with stroke, while the loss of DALYs increased by 143% compared to that in 1990 (1). Complications after stroke are not only common but also cause devastating effects on stroke recovery, leading to poor outcomes (4–6). Therefore, in addition to life-saving acute stroke treatment, optimal strategies to minimize complications and enhance stroke recovery in stroke survivors are critical as well.

Stroke is often associated with impairment in motor, sensory, or balance functions, which all predispose to fall-related injuries like fractures (7). Moreover, the accelerated loss of bone mass after stroke also contributes to fractures in stroke survivors (8, 9). Compared to subjects without stroke, patients with stroke have a 26% to 47% increased risk of fracture (10, 11). Once fractures happen in the post-stroke period, they can interfere with rehabilitation, delay functional recovery, and even lead to more complications (12, 13). One study also showed an excess in 30-day mortality in patients with stroke combined with hip fracture (14). Factors associated with post-stroke fractures include falls (15), stroke severity, and pre-stroke osteoporosis (16). Among these risk factors, osteoporosis is a highly treatable condition. With appropriate screening and pharmacological treatment (17), many post-stroke fractures can potentially be avoided.

A population-based study in Canada (18) indicated that approximately 97% of stroke survivors had an estimated 1-year fracture risk higher than 0.8% and should be screened for bone mineral density (BMD). Meanwhile, as high as 70% of stroke survivors had an estimated 1-year fracture risk greater than 2.0% and could be candidates for osteoporosis therapy regardless of their BMD. Nevertheless, according to another study on the same population (19), only 5.1% and 15.5% of stroke survivors received BMD testing and drug treatment to prevent fracture within one year post stroke, respectively. It remains unknown whether the low utilization of BMD testing and osteoporosis treatment in stroke survivors is common in other countries and healthcare systems. Therefore, using a nationwide population-based dataset, this study aimed to examine whether stroke has an impact on the utilization of BMD testing and osteoporosis treatment as well as the determinants of their utilization in stroke patients.

## Materials and methods

### Data source

We performed this retrospective cohort study using the Longitudinal Generation Tracking Database 2000 (LGTD 2000), a subset of the National Health Insurance Research Database (NHIRD), which was derived from the claims data of Taiwan’s National Health Insurance (NHI). Datasets in the NHIRD can be linked at the patient level by encrypted patient identifiers. Both the NHIRD and LGTD 2000 were described in more detail elsewhere (20). Briefly, the LGTD 2000 comprises healthcare claims data from two million beneficiaries who were randomly sampled from the NHIRD in the year 2000 and followed continuously thereafter. Individuals in the LGTD 2000 exhibit no significant differences in age, sex, or insurance premiums, as compared to those in the original NHIRD. The information of the LGTD 2000 includes demographic characteristics, outpatient and inpatient visits, diagnoses, procedures, prescriptions, and direct medical costs. Diseases and conditions were retrieved using the International Classification of Diseases 9th revision (before the end of 2015) and 10th revision (from 2016 onwards) with Clinical Modification (ICD-9-CM and ICD-10-CM) diagnosis codes. The codes used to identify diseases or conditions are listed in **Supplementary Table 1**. The validity of diagnosis codes in Taiwan’s NHIRD has been shown to be satisfactory in terms of sensitivity, specificity, and positive predictive value (21–25). Medication prescriptions were retrieved using the Anatomical Therapeutic Chemical (ATC) codes. Procedures were retrieved using the NHI billing codes.

The study protocol was approved by the Institutional Review Board of the Ditmanson Medical Foundation Chia-Yi Christian Hospital (IRB2020040). Informed consent was waived because all the study subjects were anonymized.

### Study population

**Figure 1:**
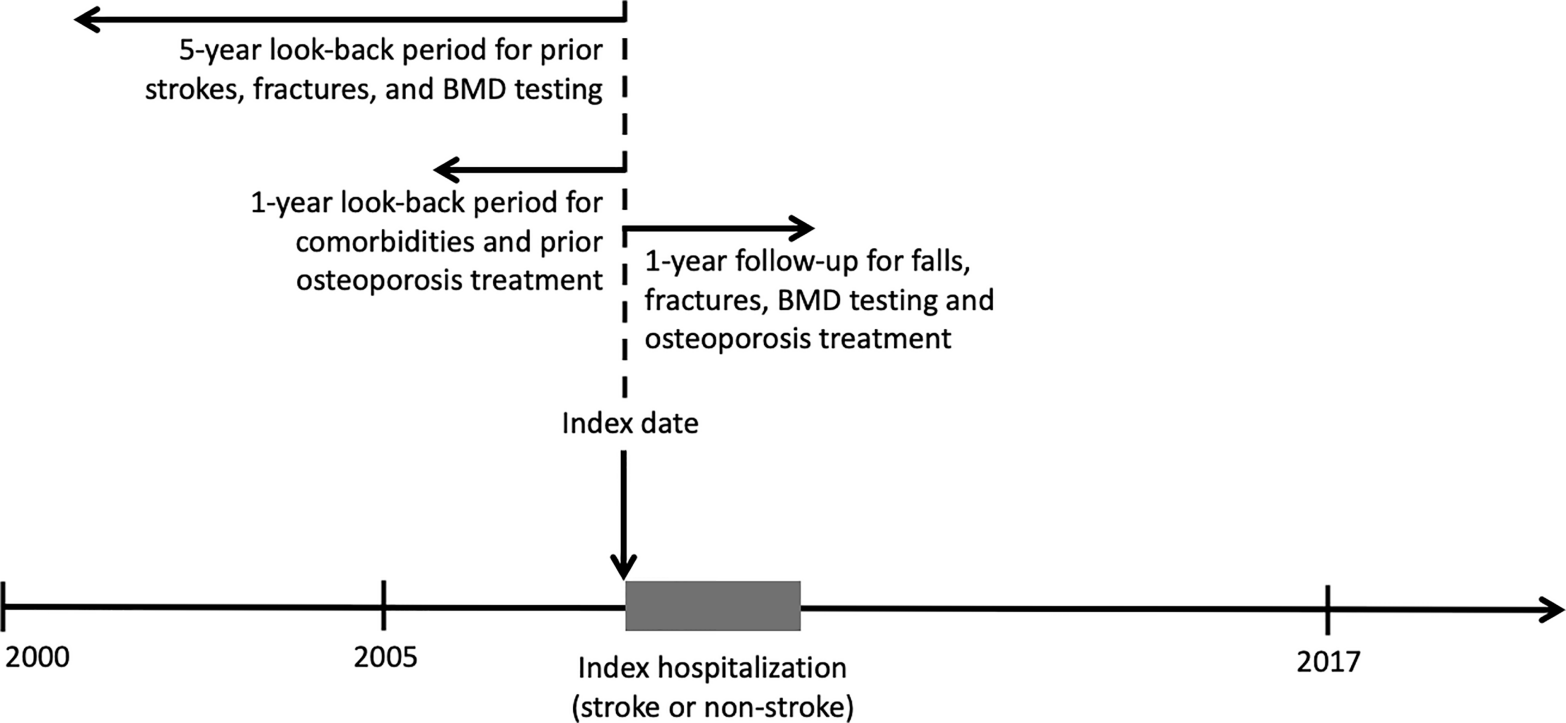
Illustration of the study design. BMD, bone mineral density; w/o, without; Tx, treatment.

**Figure 1** illustrates the study design. The study population included stroke and non-stroke cohorts. The stroke cohort consisted of patients aged 55 years and older who were hospitalized with a principal discharge diagnosis of hemorrhagic or ischemic stroke between 2005 and 2017 from the LGTD 2000. We only included those aged 55 years or above because a significant increase in the risk of post-stroke fractures was observed after this age in our previous study (16). The hospitalization was defined as the index hospitalization, and the index date was defined as the admission date of the index hospitalization. Patients were excluded if they had previous records of any type of strokes or fractures within 5 years before the index date (**Figure 1**). To evaluate the impact of stroke itself on subsequent BMD testing and osteoporosis treatment, we established the non-stroke cohort, where patients hospitalized for reasons other than stroke, fracture, or fall were drawn from the LGTD 2000 and matched to those in the stroke cohort for age ( ± 2 years), sex, and the year of hospitalization ( ± 1 year). Similarly, patients with prior strokes or fractures were excluded.

### Variables

The outcome variables were: 1. BMD testing for osteoporosis with dual-energy X-ray absorptiometry (NHI billing code 33064B) (26) and 2. osteoporosis treatment including bisphosphonate (ATC code M05BA), raloxifene (G03XC01), denosumab (M05BX04), calcitonin (H05BA), and estrogen (G03) (19) within one year after the index date (**Figure 1**).

The independent variables included age, sex, history of osteoporosis, comorbidities, prior BMD testing, prior osteoporosis treatment, as well as falls and low-trauma fractures after the index hospitalization. History of osteoporosis and comorbidities was defined as having corresponding diagnosis codes (**Supplementary Table 1**) in ≥2 outpatient visits or ≥1 inpatient visit within one year before the index date (22, 25). The comorbidities we selected included hypertension, diabetes, hyperlipidemia, atrial fibrillation, dementia, parkinsonism, and osteoporosis. Prior utilization of BMD testing within 5 years before the index date and medication prescriptions for osteoporosis within 1 year before the index date were identified using the same codes mentioned previously (**Figure 1**). Additionally, falls and fractures, including hip, upper arm, forearm, pelvis, and vertebrae after hospitalization were evaluated within 1 year after the index date (**Figure 1**). We excluded fractures related to motor vehicle accidents or high-impact trauma.

In the stroke cohort, patients’ stroke severity was estimated using the estimated National Institutes of Health Stroke Scale (eNIHSS) (27–29). Stroke severity was categorized as mild (eNIHSS ≤ 5), moderate (eNIHSS 6–13), and severe (eNIHSS >13).

### Statistical analysis

Age, sex, osteoporosis, comorbidities, and other independent variables were compared between the stroke and non-stroke cohorts using independent *t*-tests and chi-squared tests, as appropriate. We used the Fine-Gray sub-distribution hazard competing risk regression model for time-independent covariates to estimate hazard ratios (HRs) and 95% confidence intervals (CIs) for the main outcomes by considering death as a competing risk. Furthermore, falls and fractures after the index hospitalization were analyzed as time-dependent variables if the fall or fracture occurred within 1 year after the index date and before the occurrence of the main outcomes. All statistics were performed using SAS 9.4 for Windows (SAS Institute, Inc., Cary, NC). Two-tailed *p*-values less than 0.05 were considered statistically significant.

## Results

A total of 32997 stroke patients and 32997 age- and sex-matched controls comprised the stroke and non-stroke cohorts, respectively. **Table 1** lists their clinical characteristics. Stroke patients were more likely to have hypertension, diabetes, hyperlipidemia, atrial fibrillation, dementia, and parkinsonism, but were less likely to have osteoporosis and rheumatoid arthritis than controls. Stroke patients had significantly lower utilization rates of BMD testing (2.6% vs. 3.1%) and osteoporosis treatment (5.7% vs 7.6%) within 5 years and 1 year before the index date, respectively, as compared with controls. After the index hospitalization, stroke patients were more likely to have falls and low-trauma fractures than controls.

**Table 1:** Clinical characteristics of stroke patients versus non-stroke controls.

Characteristic	Total (n=65954)	Stroke (n=32977)	Non-stroke (n=32977)	*P*
Age, mean (SD)	72.0 (9.8)	72.0 (9.8)	72.0 (9.7)	0.449
55–64	18790 (28.5)	9364 (28.4)	9426 (28.6)	0.899
65–74	20869 (31.6)	10433 (31.6)	10436 (31.6)	
75–84	19611 (29.7)	9813 (29.8)	9798 (29.7)	
≥85	6684 (10.1)	3367 (10.2)	3317 (10.1)	
Female	25734 (39.0)	12867 (39.0)	12867 (39.0)	>0.999
Hypertension	43180 (65.5)	26001 (78.8)	17179 (52.1)	<0.001
Diabetes	21802 (33.1)	12778 (38.7)	9024 (27.4)	<0.001
Hyperlipidemia	17961 (27.2)	11657 (35.3)	6304 (19.1)	<0.001
Atrial fibrillation	5446 (8.3)	3865 (11.7)	1581 (4.8)	<0.001
Coronary artery disease	14033 (21.3)	6958 (21.1)	7075 (21.5)	0.266
Dementia	5336 (8.1)	3727 (11.3)	1609 (4.9)	<0.001
Parkinsonism	1755 (2.7)	1139 (3.5)	616 (1.9)	<0.001
Osteoporosis	2284 (3.5)	953 (2.9)	1331 (4.0)	<0.001
Rheumatoid arthritis	662 (1.0)	293 (0.9)	369 (1.1)	0.003
Prior BMD testing	1887 (2.9)	864 (2.6)	1023 (3.1)	<0.001
Prior osteoporosis treatment	4384 (6.6)	1886 (5.7)	2498 (7.6)	<0.001
Falls after index hospitalization	870 (1.3)	692 (2.1)	178 (0.5)	<0.001
Low-trauma fractures after index hospitalization	759 (1.2)	545 (1.7)	214 (0.6)	<0.001

Data are numbers (percentage) unless specified otherwise.

BMD, bone mineral density; SD, standard deviation.

BMD testing and osteoporosis treatment were performed in 337 (1.0%) and 1730 (5.2%) of stroke patients within 1 year after the index hospitalization, respectively, whereas these measures were performed in 254 (0.8%) and 1444 (4.7%) of controls. After adjustment was made for other variables, stroke patients were more likely to receive BMD testing (adjusted HR 1.33; 95% CI 1.11–1.58) and osteoporosis treatment (adjusted HR 1.19; 95% CI 1.11–1.29) as compared to controls (**Supplementary Table 2**).

**Table 2** shows the multivariable model for BMD testing and osteoporosis treatment after the index hospitalization in stroke patients. Older age, female sex, osteoporosis, prior BMD testing, and low-trauma fractures after stroke were positively associated with BMD testing, while severe stroke (eNIHSS >13) was negatively associated with BMD testing. Female sex, diabetes, osteoporosis, prior BMD testing, prior osteoporosis treatment, and low-trauma fractures after stroke were positively associated with osteoporosis treatment, while moderate stroke (eNIHSS 6–13), severe stroke (eNHISS >13), and dementia were negatively associated with osteoporosis treatment.

**Table 2:** Predictors of BMD testing and osteoporosis treatment among stroke patients.

	BMD Testing		Osteoporosis treatment	
	(n=337)		(n=1730)	
	Crude HR (95% CI)	Adjusted HR (95% CI)	Crude HR (95% CI)	Adjusted HR (95% CI)
Age				
55–64	1	1	1	1
65–74	2.00 (1.43–2.80)***	1.66 (1.19–2.33)**	1.14 (1.01–1.28)*	1.04 (0.92–1.17)
75–84	2.60 (1.88–3.61)***	2.16 (1.54–3.04)***	0.98 (0.86–1.11)	1.00 (0.88–1.14)
≥85	2.63 (1.76–3.93)***	2.29 (1.49–3.50)***	0.73 (0.60–0.88)**	0.94 (0.76–1.15)
Female	3.84 (3.04–4.86)***	3.09 (2.40–3.98)***	2.00 (1.82–2.20)***	1.54 (1.39–1.71)***
eNIHSS				
Mild (≤ 5)	1	1	1	1
Moderate (6–13)	0.98 (0.76–1.27)	0.87 (0.67–1.14)	0.90 (0.80–1.01)	0.85 (0.76–0.96)**
Severe (>13)	0.48 (0.36–0.65)***	0.49 (0.36–0.67)***	0.37 (0.33–0.43)***	0.39 (0.33–0.45)***
Hypertension	1.06 (0.81–1.39)	0.94 (0.71–1.24)	1.32 (1.16–1.49)***	1.03 (0.90–1.17)
Diabetes	1.02 (0.82–1.27)	0.94 (0.74–1.18)	2.90 (2.63–3.20)***	2.04 (1.83–2.27)***
Hyperlipidemia	1.35 (1.09–1.67)**	1.18 (0.94–1.49)	1.51 (1.38–1.66)***	0.98 (0.88–1.08)
Atrial fibrillation	0.82 (0.57–1.17)	0.79 (0.54–1.13)	0.71 (0.60–0.84)***	0.88 (0.74–1.05)
Coronary artery disease	0.95 (0.72–1.23)	0.88 (0.67–1.16)	1.11 (0.99–1.24)	0.98 (0.87–1.10)
Dementia	0.77 (0.53–1.12)	0.71 (0.48–1.05)	0.67 (0.56–0.79)***	0.80 (0.66–0.97)*
Parkinsonism	1.04 (0.58–1.85)	0.88 (0.48–1.60)	1.13 (0.89–1.45)	1.14 (0.88–1.48)
Osteoporosis	4.62 (3.32–6.43)***	2.29 (1.57–3.33)***	3.67 (3.11–4.32)***	1.46 (1.20–1.78)***
Rheumatoid arthritis	1.35 (0.50–3.60)	0.83 (0.30–2.27)	1.47 (0.96–2.24)	0.92 (0.58–1.48)
Prior BMD testing	4.79 (3.42–6.71)***	2.34 (1.59–3.44)***	3.12 (2.60–3.74)***	1.48 (1.21–1.80)***
Prior osteoporosis treatment	2.30 (1.66–3.19)***	1.36 (0.96–1.94)	13.97 (12.67–15.41)***	9.99 (8.93–11.18)***
Falls after stroke	9.37 (6.37–13.77)***	1.46 (0.75–2.85)	1.65 (1.27–2.14)***	1.02 (0.73–1.41)
Low-trauma fractures after stroke	17.87 (12.54–25.46)***	10.05 (5.47–18.46)***	2.56 (2.01–3.26)***	1.99 (1.43–2.77)***

* P <0.05; ** P <0.01; *** P <0.001.

BMD, bone mineral density; CI, conidence interval; eNIHSS, estimated National Institutes of Health Stroke Scale; HR, hazard ratio.

## Discussion

This nationwide population-based study showed that hospitalized stroke patients were associated with a 33% and a 19% increased probability of receiving BMD testing and osteoporosis treatment, respectively, as compared to those hospitalized for non-stroke conditions. However, the use of these two measures were quite low in both cohorts. For stroke patients, factors positively associated with both BMD testing and osteoporosis treatment included female sex, osteoporosis, prior BMD testing, and low-trauma fractures after stroke, while greater stroke severity was a negative predicting factor for the use of both measures. Older age was only positively associated with BMD testing whereas dementia was negatively associated with osteoporosis treatment. Diabetes and prior osteoporosis treatment increased the likelihood of subsequent osteoporosis treatment but not the likelihood of BMD testing post stroke.

### Stroke and fracture

In line with the results reported in the literature (10, 11, 30), stroke patients, compared to non-stroke patients, were more likely to experience falls and low-trauma fractures after the index hospitalization. Although stroke patients had a lower prevalence of prior osteoporosis and were less likely to receive BMD testing and osteoporosis treatment before the index hospitalization, the increased risk of low-trauma fractures after stroke might lead to more subsequent use of BMD testing and osteoporosis treatment. Besides, stroke patients with diabetes might be more likely to receive evaluation by endocrinologists, who are more familiar with osteoporosis treatment. Diabetes thus increased the likelihood of osteoporosis treatment in stroke patients.

On the other hand, even though stroke is a well-known risk factor for developing osteoporosis (31, 32), the utilization rates of BMD testing and osteoporosis treatment were quite low after stroke. The likelihood of using these two measures was even lower in patients with greater stroke severity. Our previous study showed an inverse association between stroke severity and the risk of post-stroke fractures (16). We speculate that patients with moderate or severe stroke were more likely to be wheelchair or bed bound and thus experienced fewer falls and fractures, which further decreased their chances of using BMD testing and osteoporosis treatment.

### Implications for insurance reimbursement

Ideally, stroke patients with a predicted risk of fracture higher than a certain threshold should undergo BMD testing and receive osteoporosis treatment if indicated, regardless of having a history of fracture or not (19, 33). However, in the context of limited medical resources, real-world clinical practice is typically dictated by the complex interactions between patient characteristics, physician judgement, and the policy of the healthcare system. According to the current regulations of Taiwan’s NHI (https://www.nhi.gov.tw), BMD testing and osteoporosis treatment were mainly reimbursed for the elderly and post-menopausal women. No wonder this study found that older age was positively associated with BMD testing while female sex was associated with BMD testing and osteoporosis treatment.

On the other hand, stroke patients have a substantial risk of fractures. The incidence of any fracture in those with first-ever stroke was estimated to be 2.40%, 5.54%, and 8.03% at 1, 3, and 5 years post stroke, respectively (16). This study further demonstrated that stroke patients carried a significantly higher risk of low-trauma fracture within one year than non-stroke patients (1.7% vs. 0.6%, p <0.001). In particular, stroke may contribute more to the risk of subsequent osteoporosis in the male sex and younger age (31). A decline in cognitive function and a high comorbidity burden such as dementia also increase the risk of osteoporotic fractures among stroke survivors (34). Since insurance reimbursement regulations may have a significant impact on the use of osteoporosis treatment and even affect patient outcomes (26), the reimbursement policy for the use of BMD testing and osteoporosis treatment should be more generous in stroke patients, especially in men, young patients, and those with dementia, to prevent such an untoward complication. Nevertheless, the cost-effectiveness of these fracture prevention strategies in stroke patients still needs more studies to inform future policy making.

### Implications for clinical practice

Although the proportion of receiving osteoporosis treatment decreased from 5.7% to 5.2% after the index hospitalization in patients with stroke, the proportion of this measure decreased even more (from 7.6% to 4.7%) in patients hospitalized for non-stroke conditions. These findings might echo the observation made by Kapoor et al. (19) that, not only stroke, but also hospitalization per se, could affect the persistence of osteoporosis treatment. The persistence of osteoporosis treatment in the real world is known to be poor (35). A systematic review of real-world evidence showed that hospitalization is one of the determinants of the persistence with oral bisphosphonates for osteoporosis (36). As compared to those hospitalized for non-stroke conditions, the impact of hospitalization on the persistence of osteoporosis treatment may be smaller in patients hospitalized for stroke because the higher prevalence of low-trauma fractures may have prompted BMD testing and osteoporosis treatment. Nevertheless, osteoporosis and associated risk of fracture are not self-limited, and thereby life-long management is generally required (33). Moreover, in addition to fracture prevention, osteoporosis treatment may confer additional clinical benefits. For example, bisphosphonate use was associated with reduced all-cause mortality among patients with osteoporosis (37). For patients who have already received osteoporosis treatment before hospitalization, more efforts are needed to improve the persistence of this measure, irrespective of whether they have stroke or not. Furthermore, for stroke patients who are treatment-naïve, validated fracture risk scores (18) can be implemented to select high-risk patients for BMD testing and osteoporosis treatment if indicated. Finally, more randomized clinical trials are warranted to provide high-quality evidence regarding primary prevention of fractures with anti-osteoporosis drugs.

### Limitations

This study has several limitations. First, because of the restrictive Taiwan’s NHI regulations, patients might pay out-of-pocket to receive BMD testing and osteoporosis treatment. Such information was not available in the database used for this study. Consequently, the utilization rates of both measures were likely to be underestimated. Second, some factors that are known to affect the persistence of osteoporosis treatment, such as tobacco use, marital status, and educational level (38, 39), were also unavailable in the database. Since these factors were likely to be associated with stroke, the relationship between stroke and the persistence of osteoporosis treatment could be biased without adjusting for these factors. Third, a BMD T-score of less than -2.5 is required for reimbursement of osteoporosis treatment according to Taiwan’s NHI regulations after 2011 (26). Some of the study patients might discontinue their osteoporosis treatment because of this reimbursement regulation. Since the results of BMD testing are lacking in claims data, we were unable to determine how this reimbursement regulation had impacted the use of osteoporosis treatment. Nonetheless, this factor was likely to be non-differential for patients with and without stroke and should not have affected the estimates in this study. Finally, although adjustments have been made for a variety of comorbidities such as parkinsonism in the multivariable model, residual confounders might still exist.

## Conclusions

The utilization rates of BMD testing and osteoporosis treatment were low among stroke survivors, even though patients hospitalized for stroke had a higher chance of receiving BMD testing and osteoporosis treatment than those hospitalized for non-stroke conditions. Patients with greater stroke severity were particularly less likely to access these osteoporosis preventive measures. In light of the substantial risk of fracture, effective strategies to improve the quality of osteoporosis care in stroke survivors are urgently needed to be developed.

## Data availability statement

The data analyzed in this study is subject to the following licenses/restrictions: Taiwan’s NHIRD is maintained and regulated by the Health and Welfare Data Science Center at the Ministry of Health and Welfare in Taiwan. The dataset only could be utilized in the division of the Health and Welfare Data Science Center. Researchers who are interested to analyze this dataset can request access to the Taiwan Ministry of Health and Welfare. Requests to access the datasets should be directed to Taiwan Ministry of Health and Welfare. Requests to access these datasets should be directed to https://dep.mohw.gov.tw/DOS/cp-5119-59201-113.html.

## Ethics statement

The studies involving human participants were reviewed and approved by Institutional Review Board of the Ditmanson Medical Foundation Chia-Yi Christian Hospital (IRB2020040). Written informed consent for participation was not required for this study in accordance with the national legislation and the institutional requirements.

## Author contributions

All authors had full access to all the data in the study and take responsibility for the integrity of the data and the accuracy of the data analysis. Study concept and design: C-HH, C-YH and S-FS. Acquisition of data: W-TH. Analysis and interpretation of data: All authors. Drafting of the manuscript: C-HH, S-FS and C-YH. Critical revision of the manuscript for important intellectual content: All authors. Study supervision: C-YH. All authors contributed to the article and approved the submitted version.

## Funding

This research was funded by the Ditmanson Medical Foundation Chia-Yi Christian Hospital Research Program [grant number R109-013]. The funder of the research had no role in the design and conduct of the study, interpretation of the data, or decision to submit for publication.

## Acknowledgments

The authors thank the help from the Health and Welfare Data Science Center of Taiwan’s Ministry of Health and Welfare for maintaining and processing the data, as well as facilitating the extraction of data. This study is based in part on data from the National Health Insurance Research Database provided by Taiwan’s Ministry of Health and Welfare. The interpretation and conclusions contained herein do not represent the position of Taiwan’s Ministry of Health and Welfare. The authors also thank Ms. Li-Ying Sung for English language editing.

## Conflict of interest

The authors declare that the research was conducted in the absence of any commercial or financial relationships that could be construed as a potential conflict of interest.

## Publisher’s note

All claims expressed in this article are solely those of the authors and do not necessarily represent those of their affiliated organizations, or those of the publisher, the editors and the reviewers. Any product that may be evaluated in this article, or claim that may be made by its manufacturer, is not guaranteed or endorsed by the publisher.
