# Supplementary material for: Utilization of screening and treatment for osteoporosis among stroke survivors
Source: Front Endocrinol (Lausanne). 2022 Dec 1;13:1043863. doi: 10.3389/fendo.2022.1043863 (PMC9751409; doi:10.3389/fendo.2022.1043863)
Supplement: Supplementary file 1 [file DataSheet_1.docx]

Supplementary Materials

**Supplementary Table 1.** Diagnosis codes for identifying diseases or conditions.

| Disease or condition | ICD-9-CM | ICD-10-CM |
| --- | --- | --- |
| Ischemic stroke | 433, 434 | I63 |
| Hemorrhagic stroke | 430, 431 | I60, I61 |
| Any type of stroke | 430 to 434, 436, and 438 | I60 to I63, I65 to I67, and I69 |
| Any fractures | 800 to 829 | S02, S62.2, S62.3, S62.5, S62.6, S62.0, S62.1, S12, S220, S221, S320, M484, M485, S72.0, S72.1, S422 to S499, and S52 |
| Falls | E880 to E888 | W00 to W19 |
| Hypertension | 401, 402, 403, 404, and 405 | I10 and I15 |
| Diabetes | 250 | E08 to E11, E13 |
| Hyperlipidemia | 272.0, 272.1, 272.2, 272.3, 272.4, and 272.9 | E78.0, E78.1, E78.2, E78.3, E78.4, E78.5 and E78.9 |
| Atrial fibrillation | 427.31 | I48.0, I48.2 and I48.91 |
| Coronary artery disease | 410, 411, 412, 413 and 414 | I21, I24 and I25 |
| Dementia | 290, 294 and 331 | G30, F00, F01, F02 and F03 |
| Parkinsonism | 332 | F02.3, G20, G21.0 to G21.4, G21.8, G21.9 and G22 |
| Osteoporosis | 733 | M80 to M82 |
| Rheumatoid arthritis | 714 | M05, M06 |

ICD-9-CM: International Classification of Diseases, 9th revision, with clinical modification; ICD-10-CM: International Classification of Diseases, 10th revision, with clinical modification.

| **Supplementary Table 2.** Predictors of BMD testing and osteoporosis treatment among all patients. | | | | | | | |
| --- | --- | --- | --- | --- | --- | --- | --- |
|  | BMD | | |  | Osteoporosis treatment | | |
|  | (n=591) | | |  | (n=3174) | | |
|  | Crude HR (95% CI) |  | Adjusted HR (95% CI) |  | Crude HR (95% CI) |  | Adjusted HR (95% CI) |
| Stroke vs. non–stroke | 1.33 (1.13–1.56)*** |  | 1.33 (1.11–1.58)** |  | 1.20 (1.12–1.29)*** |  | 1.19 (1.11–1.29)*** |
| Age |  |  |  |  |  |  |  |
| 55–64 | 1 |  | 1 |  | 1 |  | 1 |
| 65–74 | 1.87 (1.47–2.38)*** |  | 1.54 (1.20–1.96)*** |  | 1.06 (0.97–1.15) |  | 0.91 (0.84–1.00) |
| 75–84 | 2.13 (1.68–2.70)*** |  | 1.66 (1.29–2.12)*** |  | 0.86 (0.78–0.94)*** |  | 0.77 (0.70–0.85)*** |
| ≥ 85 | 1.94 (1.43–2.65)*** |  | 1.50 (1.08–2.06)* |  | 0.61 (0.52–0.71)*** |  | 0.64 (0.54–0.74)*** |
| Female | 4.75 (3.94–5.73)*** |  | 3.70 (3.03–4.52)*** |  | 2.36 (2.20–2.54)*** |  | 1.73 (1.60–1.87)*** |
| Hypertension | 1.23 (1.04–1.47)* |  | 0.96 (0.79–1.16) |  | 1.51 (1.40–1.64)*** |  | 1.15 (1.05–1.25)** |
| Diabetes | 1.13 (0.96–1.34) |  | 0.98 (0.82–1.18) |  | 2.55 (2.38–2.73)*** |  | 1.71 (1.58–1.84)*** |
| Hyperlipidemia | 1.45 (1.23–1.72)*** |  | 1.31 (1.09–1.58)** |  | 1.83 (1.70–1.96)*** |  | 1.27 (1.18–1.38)*** |
| Atrial fibrillation | 0.76 (0.55–1.06) |  | 0.64 (0.46–0.89)** |  | 0.72 (0.62–0.83)*** |  | 0.76 (0.65–0.88)*** |
| Coronary artery disease | 0.93 (0.76–1.14) |  | 0.87 (0.71–1.08) |  | 1.08 (0.99–1.17) |  | 1.00 (0.91–1.09) |
| Dementia | 0.92 (0.67–1.24) |  | 0.72 (0.52–0.99)* |  | 0.65 (0.55–0.75)*** |  | 0.67 (0.57–0.79)*** |
| Parkinsonism | 0.89 (0.52–1.51) |  | 0.79 (0.46–1.35) |  | 0.97 (0.78–1.21) |  | 1.12 (0.89–1.40) |
| Osteoporosis | 5.10 (4.08–6.39)*** |  | 2.52 (1.94–3.26)*** |  | 3.79 (3.39–4.23)*** |  | 1.54 (1.35–1.76)*** |
| Rheumatoid arthritis | 1.88 (1.04–3.41) |  | 1.11 (0.60–2.04) |  | 1.40 (1.04–1.89)* |  | 0.85 (0.62–1.15) |
| Prior BMD testing | 5.55 (4.40–7.00)*** |  | 2.52 (1.93–3.29)*** |  | 3.03 (2.66–3.45)*** |  | 1.22 (1.05–1.41)** |
| Prior osteoporosis medication | 2.31 (1.83–2.91)*** |  | 1.27 (0.98–1.63) |  | 14.30 (13.33–15.34)*** |  | 10.62 (9.80–11.50)*** |
| Falls after admission | 11.37 (8.21–15.73)*** |  | 1.52 (0.86–2.68) |  | 1.73 (1.37–2.19)*** |  | 0.96 (0.69–1.34) |
| Low–trauma fractures after admission | 20.21 (15.18–26.90)*** |  | 11.41 (6.92–18.81)*** |  | 2.74 (2.23–3.36)*** |  | 2.46 (1.78–3.40)*** |
| * *P* <0.05; ** *P* <0.01; *** *P* <0.001. | | | | | | | |
| BMD, bone mineral density; CI, confidence interval; HR, hazard ratio. | | | | | | | |
